# The evolutionary tale of eight novel plasmids in a colistin-resistant environmental *Acinetobacter baumannii* isolate

**DOI:** 10.1099/mgen.0.001010

**Published:** 2023-05-12

**Authors:** Farzana T. Prity, Liam A. Tobin, Ram Maharajan, Ian T. Paulsen, Amy K. Cain, Mehrad Hamidian

**Affiliations:** ^1^​ ARC Centre of Excellence in Synthetic Biology, School of Natural Sciences, Macquarie University, Sydney, NSW, 2109, Australia; ^2^​ Australian Institute for Microbiology & Infection, University of Technology Sydney, Ultimo, NSW, 2007, Australia

**Keywords:** *Acinetobacter baumannii*, colistin resistance, *mcr-4.7*, plasmid, environmental, Tn*6926*, Transposon

## Abstract

*
Acinetobacter baumannii* is an important opportunistic pathogen known for its high levels of resistance to many antibiotics, particularly those considered last resorts such as colistin and carbapenems. Plasmids of this organism are increasingly associated with the spread of clinically important antibiotic resistance genes. Although *A. baumannii* is a ubiquitous organism, to date, most of the focus has been on studying strains recovered from clinical samples ignoring those isolated in the environment (soil, water, food, etc.). Here, we analysed the genetic structures of eight novel plasmids carried by an environmental colistin-resistant *A. baumannii* (strain E-072658) recovered in a recycled fibre pulp in a paper mill in Finland. It was shown that E-072658 carries a new variant of the *mcr-4* colistin resistance gene (*mcr-4.7*) in a novel Tn*3-*family transposon (called Tn*6926*) carried by a novel plasmid p8E072658. E-072658 is also resistant to sulphonamide compounds; consistent with this, the *sul2* sulphonamide resistance gene was found in a p*dif* module. E-072658 also carries six additional plasmids with no antibiotic resistance genes, but they contained several p*dif* modules shared with plasmids carried by clinical strains. Detailed analysis of the genetic structure of all eight plasmids carried by E-072658 showed a complex evolutionary history revealing genetic exchange events within the genus *Acinetobacter* beyond the clinical or environmental origin of the strains. This work provides evidence that environmental strains might act as a source for some of the clinically significant antibiotic resistance genes.

## Data Summary

Complete genome assemblies of *Acinetobacter baumannii* E-072658 including its chromosome and eight plasmids p1E072658, p2E072658, p3E072658, p4E072658, p5E072658, p6E072658, p7E072658 and p8E072658 are available in the GenBank non-redundant database under GenBank accession numbers CP061705, CP061713, CP061712, CP061711, CP061710, CP061709, CP061708, CP061707 and CP061706, respectively.

**Impact Statement**

*
Acinetobacter baumannii* has become a globally significant opportunistic pathogen that is difficult to treat given its high levels of resistance to the last line of antibiotic defence, such as colistin and carbapenems. Hence it is imperative to understand how resistance emerges, evolves and spreads through *A. baumannii* populations. However, understating the evolution of resistance has largely been hampered by the fact that most studies have focused on clinical strains, which are often highly clonal. This ignores the possibility that the source for some of the clinically significant resistance genes might be in the environment that ultimately accumulate in clinical strains. Here, we studied the evolution of eight plasmids carried by an environmental strain (E-072658) and showed that one of the plasmids carries a novel variant of the *mcr-4* colistin resistance gene, which confers resistance to colistin, considered a last-resort antibiotic. This novel variant of the *mcr-4* gene (*mcr-4.7*) might be the origin of the variants of the *mcr-4* genes that are found in clinical strains. Moreover, this study showes several links of specific modules found in other plasmids of E-072658 to their closely related homologues of plasmids in clinical strains, providing further evidence of genetic exchange events between clinical and environmental strains. This study calls for further investigation of environmental strains to reveal the intricacies of the origin and evolution of antibiotic resistance.

## Introduction

*Acinetobacter baumannii* is an opportunistic Gram-negative pathogen that has attracted attention given its ability to develop resistance to many antibiotics [1, 2]. *A. baumannii* causes a wide range of nosocomial infections, including pneumonia, bacteraemia, and urinary tract and wound infections [1]. Indeed, the spread of *A. baumannii* strains resistant to all commercially available antibiotics, including those considered last resort (e.g. colistin and carbapenems) [3, 4], has become a significant global problem [3, 5]. This is recognized by the World Health Organisation, deeming carbapenem-resistant *A. baumannii* as the number one priority for antibiotic development [6]. Although *A. baumannii* strains are mainly associated with clinical settings, they have also been routinely identified in a wide range of non-clinical environments, including food, water, sewage and soil, as well as from pig and cattle faecal samples [7–15]. Therefore, it has recently been suggested that *A. baumannii* is a One Health problem (with the environment, humans and animals considered interconnected), indicating a need for a transdisciplinary research approach to study *A. baumannii* strains isolated from both clinical environmental sources (soil, water, food, etc.) [16].

In *A. baumannii *, the spread of antibiotic resistance is known to occur mainly by the horizontal acquisition of antibiotic resistance genes (ARGs) primarily via mobile genetic elements (MGEs) such as transposons and plasmids [3, 17–19]. In *A. baumannii,* most antibiotic-resistance genes are often in chromosomal genomic islands [3, 17, 18]. However, plasmids are also increasingly recognized as a significant mode for disseminating antibiotic-resistance genes, such as those conferring resistance to the front-line carbapenems and colistin antibiotics [20–23]. *A. baumannii* has a unique repertoire of plasmids that capture and mobilize a wide range of genetic material involved in pathogenesis and antibiotic resistance [20, 21, 24] that has recently been classified [25]. Most comparative genomics studies have focused on nosocomial strains, leaving the evolution, origin and genetic structure of plasmids carried by environmental strains poorly understood. Here, we analysed the genetic structure of eight plasmids carried by E-072658, an environmental *A. baumannii* strain recovered in recycled fibre pulp in a paper mill in Finland. This study shows that all eight plasmids carried by E-072658 are novel, including one that carries a novel variant of the *mcr-4* colistin resistance gene [4], and have a complex evolutionary history and share common origins with those carried by clinical strains. Furthermore, only recently characterized, p*dif* modules have been uncovered in *Acinetobacter* plasmids where they comprise pairs of Xer recombination sites called plasmid*–dif* (p*dif*) sites that flank a gene or genes [26, 27]. These p*dif* sites are remarkable for two reasons. First, p*dif* modules have been isolated from plasmids of bacteria in permafrost cores, and many gene types, including ARGs and heavy metal resistance genes, are located in p*dif*-modules in plasmids. These p*dif*-modules assist resistance genes that they carry to move between different plasmids. Second, p*dif* sites flank carbapenem resistance genes [e.g. *tet39, mph-msr(E), oxa58* and *oxa24*] in major global clones of *A. baumannii* [26, 27]. However, little is known about the distribution of p*dif* modules in *Acinetobacter* plasmids from different environmental niches. Here, p*dif* modules found on E-072658 plasmids were identified, analysed and tracked.

## Methods

### Bacterial genome sequence

Sequences of eight novel plasmids previously reported in the genome sequence of *A. baumannii* strain E-072658 [28], recovered in recycled fibre pulp in a Finnish paper mill, and Hamidian *et al*. [29] were analysed in this study.

### Antibiotic resistance profile

The antibiotic resistance profile against 23 antibiotics was previously determined [29] using the standard CDS (http://cdstest.net) disc diffusion method. Here, we measured the minimal inhibitory concentration (MIC) against colistin using the standard micro broth dilution method as previously described [30, 31] and interpreted according to the Clinical and Laboratory Standards Institute (CLSI) guidelines for *Acinetobacter* spp*.* [32].

### Bioinformatics and sequence analysis

The resistance genes and insertions sequences were manually annotated using ResFinder (https://cge.cbs.dtu.dk/services/ResFinder/) and ISFinder (https://www-is.biotoul.fr/), respectively. Protein coding regions were characterized using blastp (https://blast.ncbi.nlm.nih.gov/Blast.cgi?PAGE=Proteins), Pfam (http://pfam.xfam.org/) and UniProt (https://www.uniprot.org/) searches. Standalone blast (www.ncbi.nlm.nih.gov/books/) was used to describe the structure of plasmids carried by E-072658. A combination of manual sequence analysis and a local database of known p*dif* sites [26, 27] that was curated here were used to identify p*dif* modules in E-072658 plasmids. Putative plasmid replication initiation proteins were typed (and named) using the *Acinetobacter* Plasmid Typing (APT) scheme publicly available at GitHub (https://github.com/MehradHamidian/AcinetobacterPlasmidTyping) [25]. The CRISPR (clustered regularly interspaced short palindromic repeats) sequences were explored using the CRISPRCasFinder webtool available at https://crisprcas.i2bc.paris-saclay.fr/CrisprCasFinder/Index. The SnapGene (V6.0.5) software manually annotates regions of interest and draws figures to scale using the Illustrator (v26.2.1) program.

### Mating assays

Recipients *Escherichia coli* K-12 (BW25113) and *A. baumannii* (ATCC 17978) were grown in cation adjusted Mueller Hinton (CAMH) broth without antibiotics. For conjugation experiments, *A. baumannii* strain E-072658 was grown in CAMH (Becton Dickinson) broth supplemented with 10 µg ml^−1^ colistin (Sigma-Aldrich) overnight at 37 °C with shaking at 200 r.p.m. Mating assays were performed by mixing the same volume of donor and recipient strains (a total of 100 µl of the overnight cultures) after washing with PBS followed by spotting on CAMH agar plates and incubating overnight at 37 °C. Cells were resuspended and diluted in PBS, and transconjugants were selected by plating on CAMH agar plates containing rifampicin (100 mg l^−1^) and colistin (5 mg l^−1^). Transconjugants were further tested for rifampicin resistance to which recipient cells were resistant to differentiate them from spontaneous rifampicin mutants of E-072658 by targeted PCR. For PCR tests, primer sets MH68 (5′-CAGATTTGCCCAAAGATGGT-3′) and MH69 (5′-CGATGATAAGCACGAGCAGA-3′), and mcr4.7F (5′-CCAGCATTGGTACGCTAGTT-3′) and mcr4.7R (5′-TCGTTGGCATTGGGATAGTC-3′) were used. The primer set MH68/69 was designed to target *comM* in E-072658, which is interrupted (by Tn*6021*) in the ATCC 17978 strain. The primer set mcr4.7F/R used for detecting the *mcr4.7* gene in E-072658.

## Results and discussion

### *A. baumannii* E-072658 carries eight novel plasmids

We previously reported that the environmental *A. baumannii* strain E-072658 carries eight plasmids [29]. As also previously reported, E-072658 belongs to a novel sequence type (ST649) and it is not related to any other strains characterized to date (including all clinical and environmental strains). Its genome also contains over 440 insertion sequences, suggesting that it might have a defective CRISPR/cas system. The CRISPR/cas system is an adaptive immune system of bacteria and archaea against exogenous DNA. In fact, the CRISPR/cas system protects the bacteria from invaders, including MGEs or bacteriophages [33]. Here, our analysis showed that the E-072658 chromosome does not contain a CRISPR/cas locus, consistent with the accumulation of over hundreds of insertion sequences in its genome combined with eight plasmids.

Here, we dissected the genetic structures of all eight plasmids and examined their evolutionary relationship with other plasmids publicly available in GenBank. Analysis performed here showed all plasmids carried by E-072658 are novel given their differences to other known *A. baumannii* plasmid types. Specific modules found on the E-072658 plasmids were also tracked and revealed several potential exchange events with plasmids carried by clinical strains.

### p8E072658, a novel large Rep_3 family plasmid carrying the *mcr-4.7* colistin resistance gene, in a Tn*3*-family transposon

**Fig. 1.**
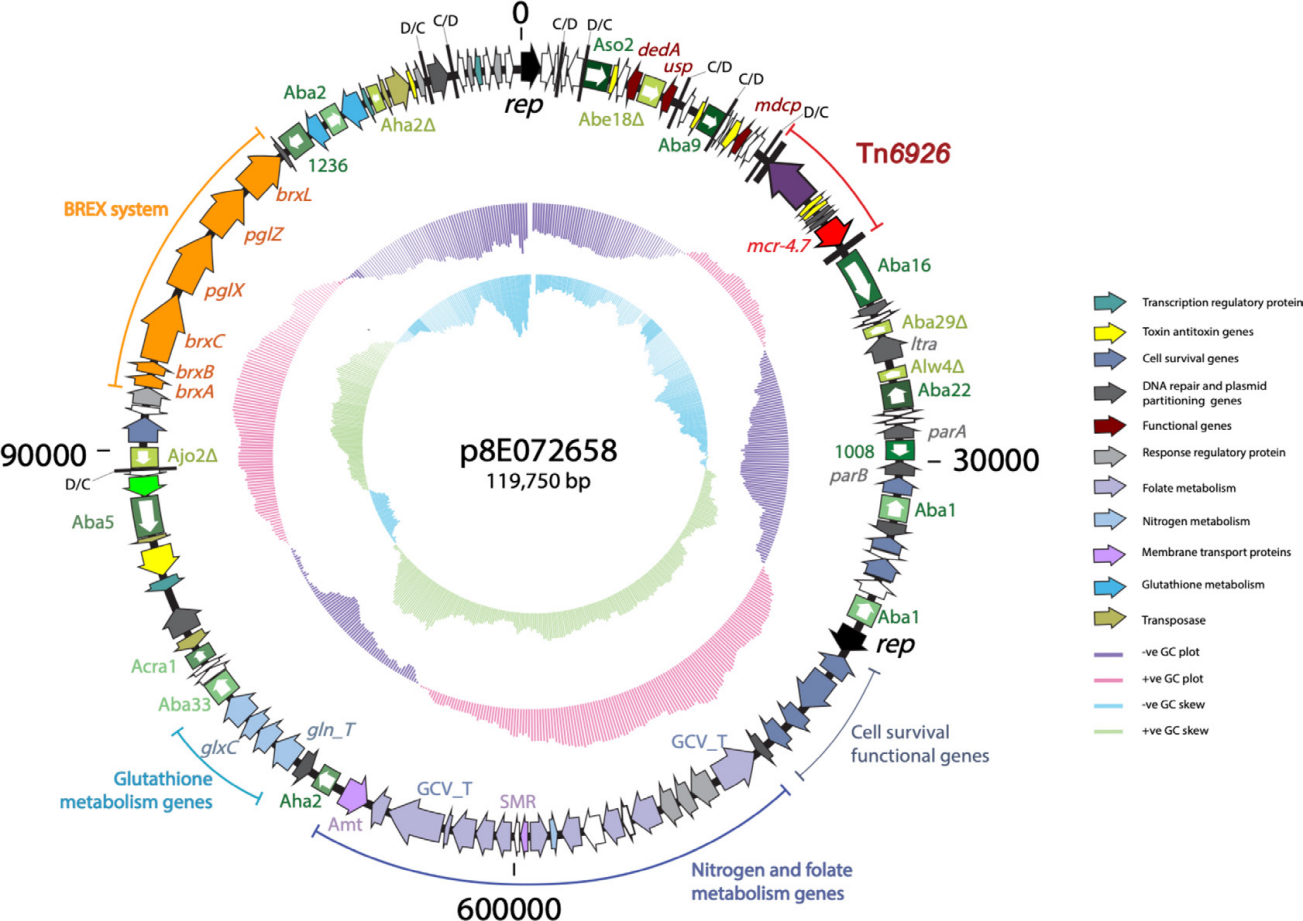
Circular representation of p8E072658, showing the distribution of functional genes. The outer black ring represents the backbone of the plasmid. Arrows indicate the extent and orientation of genes and are colour coded as per their associated functions with the keys shown on the right. The boxed arrows are insertion sequences (IS) and are labelled along the IS elements. The two inner rings represent the GC plot and GC skew, respectively. Small vertical black bars labelled C/D or D/C represent the p*dif* recombination sites (XerC/D or XerD/C).

p8E072658 is 119 750 bp and the largest plasmid carried by E-072658 (Fig. 1). p8E072658 encodes two novel putative replication initiation proteins (Rep), which belong to the Rep_3 superfamily (Pfam01051), representing the most abundant Rep type in *A. baumannii* plasmids [25]. These Rep proteins were named R3-T73 (locus id: H2787_17600) and R3-T74 (locus id: H2787_17845) and included in the recently developed APT scheme [25]. The closest match to *r3-T73* (locus id: H2787_17600) is *r3-T45* (GenBank accession number CP044520.1) while H2787_17845 does not share any DNA identity with other known *rep* sequences.

p8E072658 carries a novel allele of the *mcr-4* colistin resistance gene named *mcr-4.7* and represents one of the first reports of an *mcr* gene being isolated from an environmental isolate. The *mcr-4*.7 gene was registered in the NCBI’s Bacterial Antimicrobial Resistance Gene Database under https://www.ncbi.nlm.nih.gov/pathogens/refgene/#mcr-4.7. Colistin is a polymyxin antibiotic and is considered a last-resort antibiotic for treating multiple antibiotic-resistant *A. baumannii* infections. However, increasing resistance levels have become a significant concern given that colistin-resistant strains are also often resistant to several or all available antibiotics [34]. The *mcr* colistin resistance genes have mainly been reported in clinical strains; hence its presence in an environmental strain is significant as it also shows its presence in the environment. The *mcr* genes phosphoethanolamine transferases modify Lipid A of the cell’s lipopolysaccharide (LPS), rendering the cells colistin resistant due to the change of the overall charge of the outer membrane. To date, ten *mcr* genes (*mcr-1* to *mcr-10*), each with several variants, have been identified worldwide in several *Enterobacterales* species (e.g. *Escherichia coli *, *Klebsiella pneumoniae* and *Salmonella *) as well as *A. baumannii* with a wide range of clinical, environmental and animal origins [35]. The *mcr-4.7* gene in p8E072658 differs from *mcr-4.1*, the first *mcr-4* allele found in *Escherichia coli* and *Salmonella* spp. [36], by four base changes, two of which are also present in *mcr-4.3* (Table 1). The four nucleotide changes cause the V179G, V236F, Q331R and V485I amino acid changes relative to Mcr-4.1 (Table 1; Fig. S1, available in the online version of this article). Resistance to colistin was tested by measuring the MIC. E-072658 was resistant to high levels of colistin (25 mg l^−1^), accounting for the presence of *mcr-4.7*. It has been shown that each *mcr* gene has different possible origins and genomic context as they can be plasmid-borne or located in the chromosome. For instance, the progenitors of *mcr-1*, *mcr-3* and *mcr-4* are *Moracella* sp., *Aeromonas* sp. and *Shewanella* sp., respectively. The *mcr-1* gene is carried in a composite transposon (Tn*6330*), flanked by two copies of ISApl1, and was first identified on an Incl2 in *E. coli* [37] while *mcr-4* was first found in an 8 kb ColE10 plasmid in *Salmonella enterica* recovered from a pig in Italy [35].

**Table 1.** Changes in nucleotide/amino acid in *mcr-4* alleles

*mcr-4* allele	First identification	Nucleotide differences compared to *mcr-4.1*	Amino acid differences compared to Mcr-4.1	References
*mcr-4.1*	*Escherichia coli, Salmonella* spp.	na	na	[36]
*mcr-4.2*	*Escherichia coli, Salmonella* Typhimurium	A992G	Gln331Arg	[47, 48]
*mcr-4.3*	* Enterobacter cloacae *	T536G, G706T	Val179Gly, Val236Phe,	[49]
*mcr-4.4*	* Escherichia coli *	C613A, A992G	Gln331Arg	[47]
*mcr-4.5*	* Escherichia coli *	C329T, A992G	Gln331Arg	[47]
*mcr-4.6*	*Salmonella keduugou*	G706T	Val236Phe,	[50]
*mcr-4.7*	* Acinetobacter baumannii *	T536G, G706T, A992G, G1453A	Val179Gly, Val236Phe, Gln331Arg, Val485Ile	This study

na, Not applicable.

**Fig. 2.**
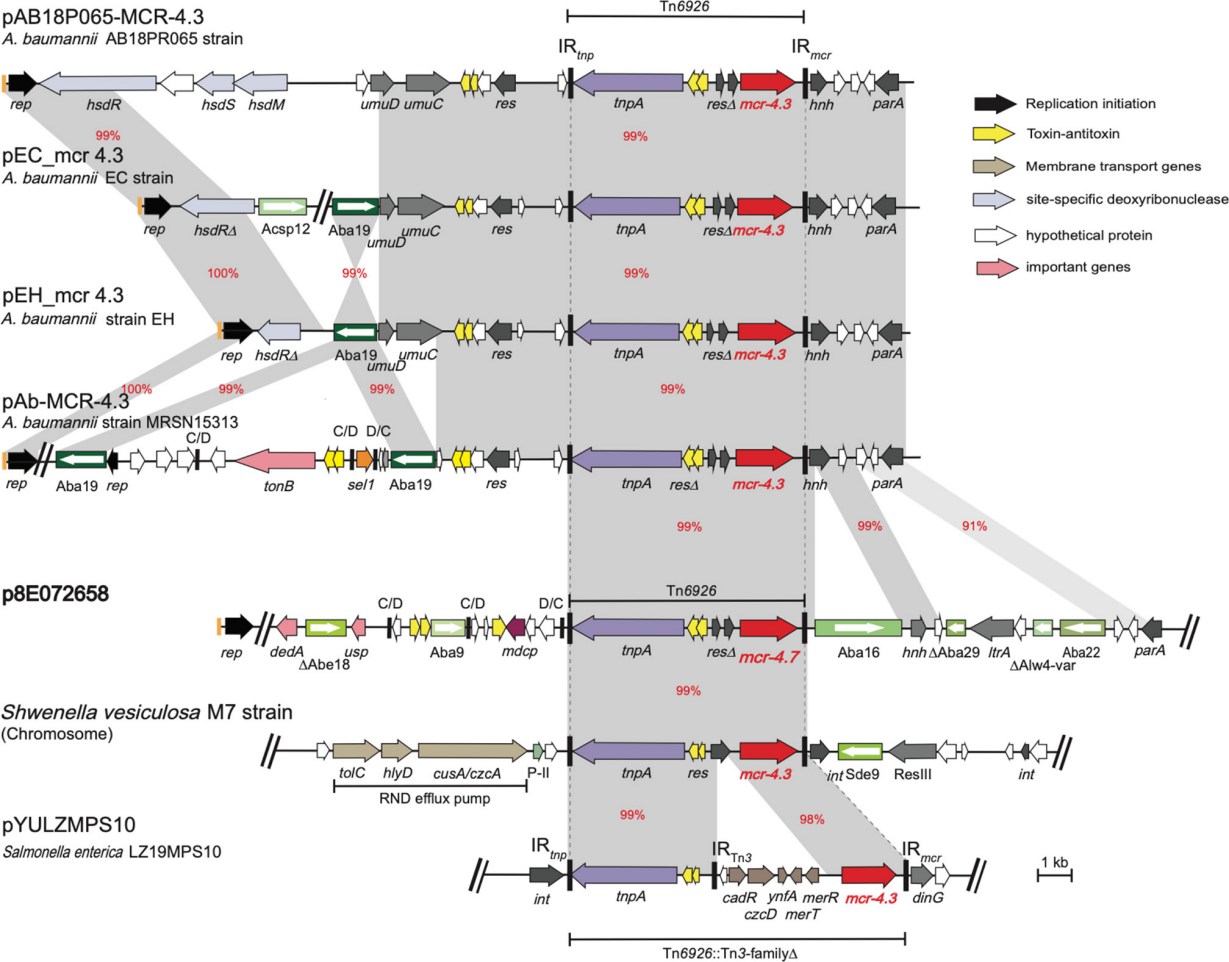
Linearized partial map of p8E072658 encoding a new allele of *mcr-4* within Tn*6926*. The horizontal central black lines represent the plasmid backbone. The arrows indicate gene orientation, and the boxed arrows represent IS. The grey shadings demonstrate significant percentage identities, marked in red. The genes/ORFs are labelled below according to their associated functions and the key is shown on the right. Iterons are shown in orange bars preceding the *rep* genes. Small vertical black bars labelled C/D or D/C represent the two p*dif* recombination sites (XerC/D or XerD/C). The long vertical bars (marked IRL and IRR) denote Tn*6926*. The scale bar is shown below and drawn to scale from GenBank accession numbers CP061706 (p8E072658), MK360916.1 (pAB18P065-MCR-4.3), CP038265.1 (pEC_mcr 4.3), CP038261.1 (pEH_mcr 4.3), CP033872.1 (pAb-MCR-4.3) and CP100354.1 (pYULZMPS10) and chromosome of *Shewenella vesiculosa* strain M7 from accession no. CP073588.1.

Later, two studies reported the presence of *mcr-4.3* in *A. baumannii* strains, recovered in clinical and pig faeces, in transposons related to Tn*3* [38, 39]. Here, analysis of the surrounding region of *mcr-4.7* indicated that this gene is in a transposon, which we named here Tn*6926*. Tn*6926* is a 6494 bp transposon with 38 bp inverted terminal repeats and encodes a TnpA and TnpR that are distantly related to the TnpAR of Tn*1-3* (with 23 and 25% amino acid identity, respectively). This, combined with the presence of a 38 bp terminal repeat, suggests that Tn*6926* also belongs to the Tn*1-3* transposon family but is distantly related to Tn*1-3*. Tn*6926* is not flanked by target site duplication. In addition to those previously described [38, 39], here we tracked and found several variants of Tn*6926* in various *A. baumannii* plasmids as well as a *Salmonella enterica* plasmid (pYULZMPS10, GenBank accession number CP100354.1; Fig. 2). It has been suggested that the genus *Shewanella* is the origin of the *mcr-4* gene [39]. A previous study showed that the *mcr-4.3* gene is in a Tn*3-*family transposon that is interrupted by another transposon (called TnShfr1) in *Shewanella frigidimarina* NCIMB 400 (GenBank accession number CP000447) [39]. However, here, we found an intact form of a variant of Tn*6926* in *Shwenella vesiculosa* strain M7 (GenBank accession number CP073588.1; Fig. 2) that confirms the genus *Shwenella* as an origin of *mcr-4*. It also showed a significant role for Tn*6926* to spread the *mcr-4* variants from *Shwenella* to *Acinetobacter *. Notably, Tn*6926* does not appear to generate target site duplications while others (in Fig. 2) are flanked by various numbers (three to five) 3–5of duplications, suggesting they do not always generate an exact number of target site duplications.

The emergence and spread of the *mcr* genes in *A. baumannii* strains is a concerning public health threat given that the wide dissemination of *mcr* from environmental to clinical samples has increased the risk of spread to humans via various routes. Prior to the emergence of *mcr* genes, colistin resistance was considered not transferable and intrinsic to the organism. Mating assays performed here did not result in conjugative transfer of p8E072658, suggesting that, under the conditions tested, p8E072658 is not conjugative, consistent with the absence of transfer functions on this plasmid. However, the finding of *mcr-4.7* in a transposon on a plasmid in an environmental *A. baumannii* strain is remarkable in many ways. Colistin is considered a last-resort antibiotic, and the spread of *mcr* genes further limits treatment options. It also shows a significant role for the environment as a source for a clinically important resistance gene, highlighting that more attention should be given to study environmental strains as the potential source of many clinically important genes.

Another striking feature of p8E072658 is the presence of 18 insertion sequences (IS) including multiple partial IS indicating a complex evolutionary history (Fig. 1). Moreover, p8E072658 encodes a BREX system (Bacteriophage Exclusion system; Fig. 1). The BREX system is a phage resistance system and has been added to the previously known mechanisms (e.g. CRISPR-Cas and restriction-modification systems) in the arsenal of bacterial defence against phages [38]. A related BREX system was found in pTol5, a 117 kb plasmid of an *Acinetobacter* sp. (GenBank accession number AP024709.1) that belongs to the diverse plasmid family of *A. baumannii* conjugative plasmids represented by pA297-3 (GenBank accession number KU744946) [23, 40]. BREX determinants were found in another *Acinetobacter* plasmid type, as recently described [41]. However, as p8E072658 is a different plasmid type this indicates that various *Acinetobacter* plasmid types carry BREX systems. p8E072658 also has a few additional regions that encode metabolic and cell survival functions (Fig. 1), suggesting possible environmental co-selection and important roles of these functions in adapting E-072658 in the environment.

### p7E072658 carries the *sul2* sulphonamide resistance gene in a p*dif* module

**Fig. 3.**
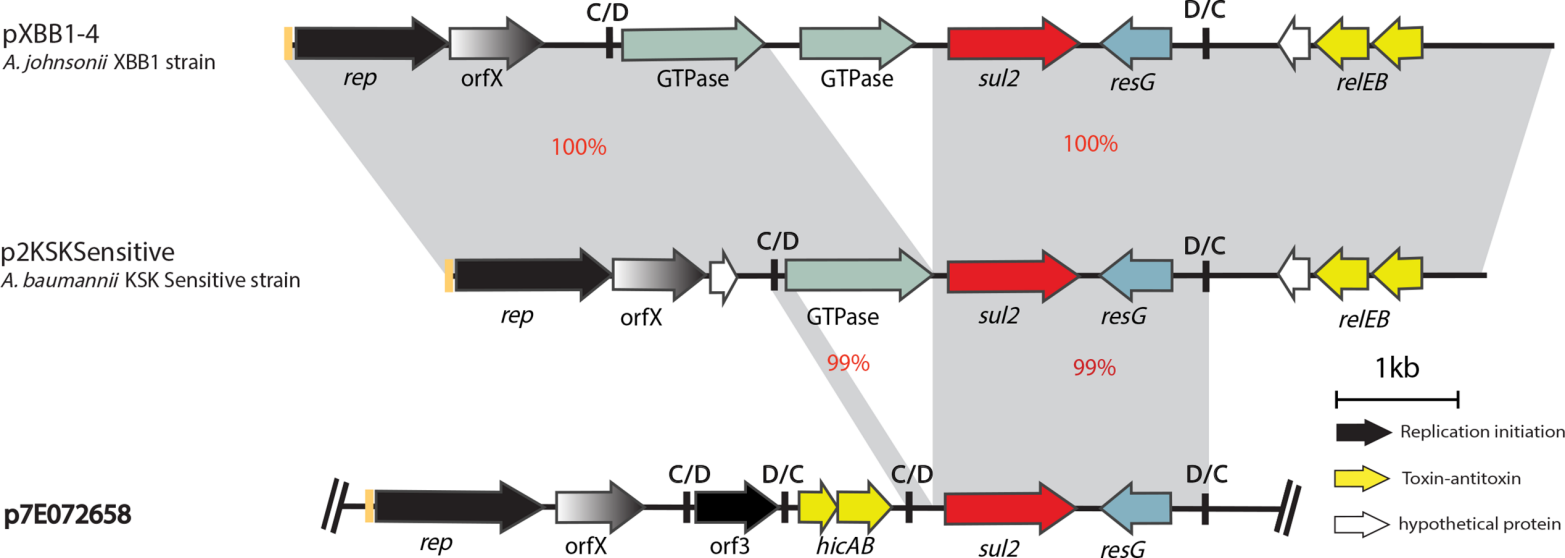
Schematic of the linearized partial map of p7E072658 containing the *sul2* gene within a p*dif* module compared to similar p*dif* modules found in unrelated plasmids. The horizontal central black lines represent the plasmid backbone. The arrows indicate gene orientation, and the boxed arrows represent IS. The grey shadings demonstrate significant percentage identities, coloured red. The genes/ORFs are labelled below according to their associated functions and the key is shown on the right. Iterons are shown in orange bars preceding the *rep* genes. Small vertical black bars labelled C/D or D/C represent the two pdif recombination sites (XerC/D or XerD/C). The scale bar is shown below and drawn to scale from GenBank accession numbers CP061707 (p7E072658), CP061707.1 (p2KSKSensitive) and CP061707.1 (pXBB1-4).

p7E072658 (GenBank accession number CP061707) is a 19 661 bp plasmid that encodes two novel Rep proteins belonging to the Rep_3 superfamily (Pfam01051), which were named R3-T75 (locus id H2787_18135) and R3-T76 (locus id H2787_18195). Closet matches to H2787_18135 and H2787_18195 are *r3-T17* (GenBank accession number CP015365) and *r3-T10* (GenBank accession number LR026972), respectively. p7E072658 carries a *sul2* sulfonamide resistance gene and the *relEB* genes encoding toxin–antitoxin functions. Like most plasmids that encode Rep_3 family Rep proteins [25], p7E072658 contains multiple p*dif* modules (Fig. 3). The *sul2* gene is often associated with the GI*sul2* genomic island [42]. Here, analysis of the *sul2* context showed that a 1222 bp segment, including *sul2* and 100 bp and 297 bp upstream and downstream of this gene, was identical to GI*sul2,* indicating a common origin and that this segment has derived from GI*sul2*. However, further analysis showed that the *sul2* gene is part of a 2699 bp long p*dif* module (Fig. 3), suggesting that it can move via spread of this module. To date, several antibiotic resistance genes have been found in p*dif* modules, but to the best of our knowledge this is the first time *sul2* has been found in this context. Here, we tracked this module and found two variants in pXBB1-4 (GenBank accession number CP061707.1) carried by *Acinetobacter johnsoni* XBB1-4 isolated in hospital sewage and p2KSKSensitive (GenBank accession number CP061707.1) carried by *A. baumannii* p2KSKSensitive recovered in a respiratory specimen, indicating spread of the *sul2* p*dif* modules across species in various sources (hospital sewage and clinical samples).

### E-072658 carries six novel cryptic plasmids containing various p*dif* modules shared with other clinical and environmental *Acinetobacter* plasmids

The remaining plasmids (p1E072658–p6E072658; Fig. S2) were cryptic. However, they encode important proteins involved in various functions such as usher protein, ABC transporter, inorganic ion transporter and toxin–antitoxin systems.

**Fig. 4.**
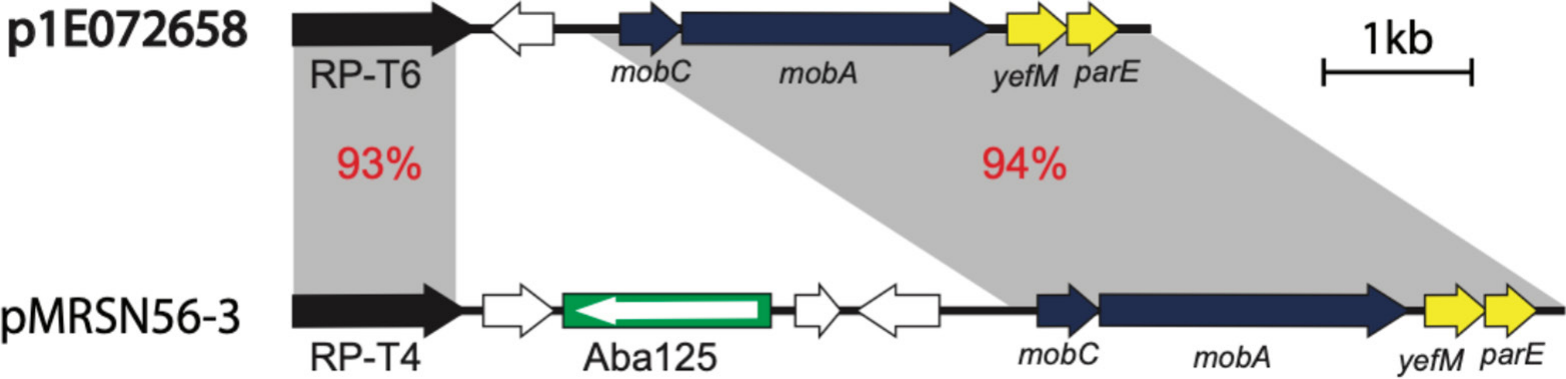
Comparison of the genetic structure of p1E072658 and pMRSN56-3. The horizontal central black lines represent the plasmid backbone. The arrows indicate gene orientation, and the boxed arrow represents IS. The grey shadings demonstrate significant percentage identities, marked in red. The genes/ORFs are labelled below according to their associated functions. The scale bar is shown below and drawn to scale from GenBank accession numbers CP061713 (p1E072658) and CP080455 (pMRSN56-2).

p1E072658 is 4483 bp in length and the only plasmid that encodes a novel putative replication protein (Locus id H2787_18615) belonging to the Rep_PriCT1 superfamily (pfam03090) (Fig. 4). The closest match to H2787_18615 is rP-T4 (GenBank accession number CM009039). This novel Rep was named RP-T78 (Table 2). Notably, most plasmids that belong to this family are large and involved in the spread of aminoglycoside and carbapenem resistance genes [25]. However, unlike other members of this diverse family, p1E072658 is cryptic. p1E072658 is a novel plasmid; however, it shares approximately 3.5 kb with pMRSN56-2 (GenBank accession number CP080455) with 93–94 % DNA identity, indicating these plasmids belong to a diverse family. Moreover, pMRSN56-2 is carried by *A. baumannii* MRSN56 recovered in 2010 from a hip infection, indicating that plasmids related to pMRSN56-2 and p1E072658 are spread across clinical and environmental samples.

**Table 2.** Properties of plasmids carried by *A. baumannii* E-072658

Plasmid name	Size (bp)	Rep type*	Locus_id	Rep family	Pfam	Mob	Pfam	p*dif* modules	IS	Important functions	GenBank Acc. no.
p8E072658	119 750	**R3-T73** **R3-T74**	**H2787_17600** **H2787_17845**	Rep-3 Rep-3	01051	–	–	8	ISAba1, IS*1008*, ISAba22, ISAlw4Δ (92%), ISAba9, ISAba29Δ, ISAba16, ISAba8, ISAbe18Δ, ISAba27Δ, IS1236, ISAba5Δ (92%), ISAcra1, ISAba33, ISAha2	Colistin resistance	CP061706
p7E072658	19 661	**R3-T75** **R3-T76**	**H2787_18135** **H2787_18195**	Rep-3 Rep-3	01051	MobA/L	03389	7	IS17∆ ISA1w14	Sulfonamide resistance	CP061707
p6E072658	18 806	R3-T36 **R3-T77**	H2787_18240 **H2787_18280**	Rep-3 Rep-3	01051	–	–	7	–	Usher protein	CP061708
p5E072658	15 545	–	na	–	–	–	–	–	ISAlw34, ISAba125 ISAha3Δ, ISaba31 ISAba27Δ, ISAba7Δ	ABC transporter	CP061709
p4E072658	7989	R3-T2	H2787_18430	Rep-3	01051	–	–	3	–	Inorganic ion transporter	CP061710
p3E07265	6763	R3-T49	H2787_18495	Rep-3	01051	MobA/L	03389	1	–	Toxin- antitoxin	CP061711
p2E072658	6018	R3-T15	H2787_18560	Rep-3	01051	MobA/L	03389	1	–	Toxin- antitoxin	CP061712
p1E072658	4483	**RP-T6**	**H2787_18615**	RepPriCT_1	03090	MobC MbeA	05713 03432	–	–	Toxin- antitoxin	CP061713

*Letters in bold type indicate novel Reps identified in this study. na, Not applicable.

p2E072658 is a 6018 bp novel plasmid. It encodes the R3-T15 replication protein (belonging to the Rep_3 superfamily, pfam01051) (locus id H2787_18560; GenBank accession number CP061712; Table 2). p2E072658 also encodes the MobA/L mobilization proteins (belonging to pfam03389) and a BrnTA toxin–antitoxin system that is widely spread in *Acinetobacter* plasmids. p2E072658 contains one p*dif* module that encodes two hypothetical proteins (ORF module). We found several plasmids that contained segments of p2E072658 with 60–83% coverage (and DNA identities ranging from 92 to 97 %). These plasmids were from different *Acinetobacter* species and various environmental sources, and included p2_010062 (GenBank accession number CP033122.1) carried by *Acinetobacter wuhouensis* WCHAW010062 recovered in sewage (with 72 % coverage and 97 % DNA identity), p9_010034 (GenBank accession number CP032277.1) found in *Acinetobacter* sp. WCHAc010034 isolated in hospital sewage (66 % coverage and ~92 % DNA identity), p2014S01-097-4 (GenBank accession number CP033554.1) in *Acinetobacter nosocomialis* strain 2014S01-097 isolated in a clinical sample (50 % coverage and 83 % identity), pXMC5x702–5k (GenBank accession number CP084309.1) in *Acinetobacter pseudolwoffii* strain XMC5x702 recovered in chicken faeces (50 % coverage and ~91 % identity) and pAS80-5 (GenBank accession number CP061548.1) in *Acinetobacter seifertii* strain AS80 isolated in human blood. Notably, most sequence homologies indicated were around the segments encoding mobilization and replication functions – again indicating a wide dissemination of these modules beyond the species and source boundaries. The ORF p*dif* module with 99 % DNA identity was also found in two *Acinetobacter* plasmids – pApW20-3 (GenBank accession number CP027661.1) and pOCUAc18-3 (GenBank accession number AP024805.1) carried by *Acinetobacter pittii* strain Ap-W20 isolated from fish and *A. baumannii* strain OCU_Ac18 isolated from a patient’s blood in Japan, respectively [43].

p3E072658 is an 6763 bp plasmid that encodes a Rep3 family (R3-T49) replication protein, a MobA mobilization protein and a toxin–antitoxin protein required for usual plasmid stabilization. p3E072658 contains a p*dif* module that encodes three hypothetical proteins, one toxin and antitoxin system and a macrodomain-containing protein. Macrodomains are highly evolutionarily conserved proteins in all three domains of life, highlighting their fundamental roles in diverse biological functions. They are known to interact with ADP-ribose metabolites, but their specific functional role in bacteria is not fully understood. However, one study showed that macrodomain proteins recognize ADP-ribosylated protein targets by bacterial exotoxin in host cells [44]. An identical module to that in p3E072658 was detected only in *A. towneri* strain SCLZS30 in plasmid p1_SCLZS30 (GenBank accession number CP090385), isolated from wastewater in China. p3E072658 and p1_SCLZS30 shared no other region, indicating that this p*dif* module can move between plasmids.

p4E072658 (7989 bp) is an R3-T2 Rep protein (formerly known as RepAci2) with no mobilization function. Plasmids encoding R3-T2 are common in ST2 strains representing global clone 2, the most widespread clinical strains globally. p4E072658 contains three p*dif* modules, one module encoding a RelE/ParE toxin–antitoxin system, flanked by two other modules encoding a SulP on the left and three hypothetical proteins on the other side.

NCBI blast searches revealed several plasmids of clinical strains with segments identical to p4E072658: for instance, the SulP transporter region of several *A. seifertii* strains isolated from human clinical samples (e.g. pAS47-2 from *A. seifertii* AS47 recovered from a blood sample in Taiwan; GenBank accession number CP061631), indicating a link between these plasmids.

To date, several *Acinetobacter* plasmids have been described that do not encode a *rep* gene, including the small plasmid pRAY and the large conjugative plasmid pA297-3 [25]. p5E072658 (15545 bp) does not encode an identifiable rep gene but contains several genes encoding ABC membrane transporter genes. p5E072658 adds yet another plasmid type that needs further investigation to reveal its replication mode.

p6E072658 is an 18806 bp plasmid that encodes two Rep3 family Rep proteins, including an R3-T36 and a novel Rep called R3-T77. The *r3-T77 rep* gene (locus id: H2787_18280) does not share significant DNA identity with any known *rep* sequence typed to date. No p*dif* module was found. p6E072658 carries the *fimD* gene*, fimC* gene and a spore coat protein U domain-containing genes. These genes (with ~98% DNA identity) were found in pXM9F202-2-186k and pAV_175–3, two plasmids in *Acinetobacter variabilis* strain XM9F202-2 (CP060812), and in plasmid AV_175 (CP078029), respectively. The *A. variabilis* strains were isolated from hospital alcohol dispensers in China.

Together, analysis of cryptic plasmids of E-072658 show the presence of several distinct p*dif* modules, and other segments that are not part of a p*dif*, shared with several *baumannii* and non-*baumannii Acinetobacter* strains recovered in clinical sources and a wide range of environmental sources. This analysis provides clear evidence for the significant role of *Acinetobacter* plasmids in spreading genetic material, beyond antibiotic resistance determinants, involved in various biological and physiological processes.

### *A. baumannii* genomes carrying multiple plasmids

To draw an overal picture of the number of plasmids carried by *A. baumannii* genomes, all complete genomes of this organism (*n*=423 as of mid-August 2022) were analysed. Analysis of these genomes showed that of the 423 genomes, 113 carried no plasmids, 273 carried three or fewer plasmids, and 35 complete *A. baumannii* genomes carried four or more plasmids (genomes with five or more in Table 3 and genomes with four plasmids in Table S1).

**Table 3.** Properties of publicly available strains that contain five or more plasmids

Strain	Date	Source	Country	ST	Plasmid name	Size (kb)	Rep	Tra	Mob	Resistance genes	Accession no.
OCU_Ac18	2016	Blood	Australia	Novel	pOCU_Ac18-1	90 090	R3-T44	–	–	–	AP024803
					pOCU_Ac18-2	12 817	R3-T6	–	MobAL	–	AP024804
					pOCU_Ac18-3	10 831	R3-T67	–	MobAL	–	AP024805
					pOCU_Ac18-4	8948	R3-T24	–	–	–	AP024806
					pOCU_Ac18-5	7104	R3-T2	–	MobAL	–	AP024807
					pOCU_Ac18-6	5049	R3-T70	–	MobA	–	AP024808
					pOCU_Ac18-7	4293	R3-T79	–	MobAL	–	AP024809
					pOCU_Ac18-8	3948	R3-T71	–	MobAL	–	AP024810
					pOCU_Ac18-9	3496	R1-T8	–	–	–	AP024811
					pOCU_Ac18-10	2444	–	–	–	–	AP024812
					pOCU_Ac18-11	2287	R3-T15	–	–	–	AP024813
E47	2013	na	Australia	1547	pE47_001	327 867	–	–	–	*mph/msr(E*), *aac(3)-lld*, *cmlB1*, *sul1*, *catB3*, *aac(6')-lb4*, *bla*_lMP-4_, *dfrA19*, *sul1*, *bla*_CARB-2_, *aphA1*	CP042557
					pE47_002	59 744	R3-T7	–	–	–	CP042558
					pE47_003	8795	R3-T66	–	MobL	–	CP042559
					pE47_004	7703	R3-T2	–	MobAL	–	CP042560
					pE47_005	5234	R3-T18	–	MobL	–	CP042561
					pE47_006	5039	–	–	MobAC	–	CP042562
					pE47_007	4715	RP-T5	–	MobC	–	CP042563
					pE47_008	3065	R3-T55	–	–	–	CP042564
					pE47_009	2427	R3-T47	–	–	–	CP042565
ACN21^a^	2018	Blood	India	85	pACN-1	116 047	R3-T3	–	–	*mph/msr(E), armA, sul1, blaCARB-2, blaOXA-420*	CP038645
					pACN-2	57 333	R3-T60	–	–	*aadB*	CP038646
					pACN-3	9909	–	–	MobC	–	CP038647
					pACN-4	9205	R3-T6	–	MobAL	*aadB*	CP038648
					pACN-5	7396	–	–	MobC	*aadB*	CP038649
					pACN-6	6944	–	–	MobC	*aadB*	CP038650
					pACN-7	5844	–	–	MobC	–	CP038651
					pACN-8	5734	–	–	MobC	*mph/msr(E), armA, sul1, blaCARB-2, bla*_OXA-420_	CP038652
E-072658	2009	Paper pulp mill	Finland	649	p8E072658	119 750	R3-T73 R3-T74	–	–	*mcr-4.7*	CP061706
					p7E072658	19 661	R3-T75 R3-T76	–	MobAL	*sul2*	CP061707
					p6E072658	18 806	R3-T5 R3-T77	–	–	–	CP061708
					p5E072658	15 545	–	–	–	–	CP061709
					p4E072658	7989	R3-T2	–	–	–	CP061710
					p3E072658	6763	R3-T49	–	MobAL	–	CP061711
					p2E072658	6018	R3-T15	–	MobAL	–	CP061712
					p1E072658	4483	RP-T6	–	MobC	–	CP061713
VB2486	2019	Sputum	India	1	pVB2486_1	99 090	RP-T1	TraLEKBVCWUNFHG	–	*bla*_OXA-23_	CP050404
					pVB2486_2	14 906	R3-T11	–	–	–	CP050405
					pVB2486_3	12 574	R3-T23	–	MobAL	*mph/msr(E*)	CP050406
					pVB2486_4	5679	–	TraD	MobAL	*aphA6*	CP050407
					pVB2486_5	5432	R3-T56	TrbL	MobAL	*aadA1*	CP050408
					pVB2486_6	2457	–	–	MobAL	–	CP050409
PM194188	2019	BAL	India	10	pPM194122_1	150 385	R3-T60	TraHY	MobL	*mph/msr(E*), *armA*, *sul1*, *cmlA5*, *arr-2*, *sul2*, *strB*, *aph(3'')-lb*, *ble*-MBL, *bla*_NDM-1_, *sul2*, *tet(B*)	CP050426
					pPM194122_2	18 783	R3-T11	–	–	–	CP050427
					pPM194122_3	7695	R3-T17	–	–	–	CP050428
					pPM194122_4	7540	R3-T1	–	–	–	CP050429
					pPM194122_5	2762	R3-T30	–	–	–	CP050430
					pPM194122_6	2419	–	–	–	–	CP050431
VB958^a^	2019	Blood	India	Novel	pVB958-1	561 419	–	–	–	–	CP040041
					pVB958-2	538 712	–	–	–	*bla*_OXA-23_, *carO*	CP040042
					pVB958-3	82 500	RP-T1	TraLEKBVCW NFHG, TrbC	–	*bla_OXA-23_*	CP040043
					pVB958-4	47 143	–	–	–	–	CP040044
					pVB958-5	16 490	R3-T11	–	–	–	CP040046
					pVB958-6	16 485	R3-T11	–	–	–	CP040045
Ab-D10a-a	2016	Spinal Fluid	Ghana	103	pAb-D10a-a_1	48 239	R3-T20	–	MobC	*dfrA1*, *mph/msr(E*), *tet39*, *aacC2d*	CP051870
					pAb-D10a-a_2	8495	R3-T10	–	–	–	CP051871
					pAb-D10a-a_3	8215	–	TraD	MobAL	*bla*_OXA-23_	CP051872
					pAb-D10a-a_4	6619	Novel	–	–	–	CP051873
					pAb-D10a-a_5	2697	R1-T3	–	–	–	CP051874
A85	2003	Sputum	Australia	1	pA85-3	86 334	RP-T1	TraALEKBVCWUNFHG, TrwBC,TrbC	–	*bla*_OXA23_	CP021787
					pA85-2	8731	R3-T1	–	–	–	CP021786
					pA85-1b	4484	–	TraD	MobA	*dfrA44*	CP021785
					pA85-1a	2726	R1-T2	–	–	–	CP021784
					pA85-1	2343	R1-T1	–	–	–	CP021783
PM193665	2019	Wound	India	10	pPM193665_1	150 385	R3-T60	TraHY	MobL	*mph/msr(E*), *armA*, *sul1*, *cmlA5*, *arr-2*, *sul2*, *strB*, *aph(3'')-lb*, *ble-MBL*, *bla*_NDM-1_, *sul2*, *tet(B*)	CP050416
					pPM193665_2	52 509	–	–	–	–	CP050417
					pPM193665_3	18 783	R3-T11	–	–	–	CP050418
					pPM193665_4	7540	R3-T1	–	–	–	CP050419
					pPM193665_5	2762	R3-T30	–	–	–	CP050420
A52	2015	Sputum	China	77	pA52-1	110 713	R3-T3	–	–	–	CP034093
					pA52-2	27 452	R3-T27	TraA	–	–	CP034094
					pA52-5	8493	R3-T24	–	–	*bla*_OXA72_	CP034097
					pA52-3	8420	–	–	–	–	CP034095
					pA52-4	3610	R3-T58	–	MobL	–	CP034096
DS002	2015	Soil	India	novel	pTS134338	134 338	R3-T7	–	–	–	CP042210
					pTS37365	37 365	–	–	–	–	CP042209
					pTS11291	11 291	R3-T2	–	MobA	–	CP042208
					pTS9900	9900	R3-T6	–	MobA	–	CP042207
					pTS4586	4586	–	–	MobA	–	CP042206

*Named here. na, Not applicable.

Plasmid incompatibility is known mainly due to two plasmids using the same replication machinery [45]. In fact, incompatibility generally occurs when copy number control elements from closely related plasmids act *in trans* to interfere with one another’s stable maintenance [46]. Almost all genomes with multiple plasmids were found to carry multiple R3-type (Rep3; Pfam01051) plasmids, suggesting they were compatible. The shared properties (the same Rep type and 122 bp iteron ~50 bp upstream of *rep*) of the Rep3 family plasmids and that there are many genomes with several Rep3 plasmid types raise the question of whether *Acinetobacter* plasmids, and in particular diverged Rep3-type plasmids, are compatible. Interestingly, no genome with more than one RP-type (encoding RepPriCT_1 type; Pfam03090) plasmid was found. While this might be coincidental, it does indicate that multiple R3-type plasmids can be stably maintained in the same cell or with other R1- or RP-type plasmids. Notably, only one strain was found (A85 recovered in Australia in 2003) that contained two R1-type (encoding R1 replication protein; Pfam01446) plasmids (GenBank accession numbers CP021783 and CP021784) (Tables 1 and S1). Although this provides clues to the incompatibility of *A. baumannii* plasmids, it does warrant extensive experimental work to establish the ability or inability of various plasmid types to be stably maintained in the same cell. Analysis of complete plasmids also showed many plasmids with no identifiable *rep* gene (Tables 1 and S1), raising the need to establish their replication mode. In addition, many small plasmids, mainly R3 type, were found to encode mobilization functions (Mob), suggesting they might be mobilizable.

## Conclusion

Resistance to last-resort antibiotics such as colistin leaves limited to no option to treat *A. baumannii* infections, given that strains causing infections in hospitals are frequently resistant to several additional antibiotics. To date, most studies have focused on analysing clinical strains, while the origin of many clinically significant resistance genes remains poorly understood. Here, we described an environmental *A. baumannii* strain recovered in recycled fibre pulp in a Finnish paper mill and showed that it carries eight novel plasmids, one containing a novel colistin resistance gene (*mcr-4.7*), which is associated with high levels of the colistin resistance phenotype. Moreover, the strain analysed here contained seven additional novel plasmids, analysis of which revealed several shared modules with *Acinetobacter* plasmids carried by clinical strains, indicating the exchange of genetic material beyond species and environmental niches. This study highlights the role of the environment, and environmental strains, as potential reservoirs for clinically significant antibiotic resistance genes and that to better understand the emergence of resistance, more attention should be given to studying and analysing strains recovered in non-clinical niches.

## Supplementary Data

Supplementary material 1
