# Supplementary material for: The evolutionary tale of eight novel plasmids in a colistin-resistant environmental Acinetobacter baumannii isolate
Source: Microb Genom. 2023 May 12;9(5):mgen001010. doi: 10.1099/mgen.0.001010 (PMC10272872; doi:10.1099/mgen.0.001010)
Supplement: Supplementary material 1 [file mgen-9-1010-s001.pdf]

Table S1. *A. baumannii* genomes containing four plasmids.

Strain Name	Date	Source	Country	ST	Plasmid Name	Size (kb)	Rep	Tra	Mob	Resistance genes carried on plasmids	GenBank Accession
AC1633	2016	Blood	Malaysia	126	pAC1633-1	174292	-	TraYHM, TrbI	-	*aac(3)-lld*, *sul2*, *bla*$_{NDM-1}$, *ble-MBL*, *aph(3'')-lb*, *strB*, *bla*$_{OXA-58}$, *mph/msr(E)*	CP059301
					pAC1633-2	12651	R3-T67	-	MobAL	*tet39*	CP059303
					pAC1633-3	9950	R3-T34	-	MobAL	-	CP059304
					pAC1633-4	5210	R3-T61	-	MobAL	-	CP059302
KSK10	2020	Respiratory	India	622	p1KSK10	218105	-	TraHY	-	*mph/msr(E)*, *armA*, *sul1*, *bla*$_{PER-7}$, *sul1*, *cmlA1*, *arr-3*, *ant(3'')-la*, *sul2*, *strAB*	CP072281
					p2KSK10	87529	RP-T1	TraLEKBVCWUNFHG	-	*bla*$_{OXA-23}$, *aphA6*	CP072282
					p3KSK10	7540	R3-T1		-	-	CP072283
					p4KSK10	5679	-	TraD	MobAL	*aphA6*	CP072284
KSK19	2020	Respiratory	India	622	p1KSK19	218105	-	TraHY	-	*mph/msr(E)*, *armA*, *sul1*, *bla*$_{PER-7}$, *sul1*, *cmlA1*, *arr-3*, *ant(3'')-la*, *sul2*, *strAB*	CP072296
					p2KSK19	87529	RP-T1	TraLEKBVCWUNFHG	-	*bla*$_{OXA-23}$, *aphA6*	CP072297
					p3KSK19	7540	R3-T1	-	-	-	CP072298
					p4KSK19	5679	-	TraD	MobAL	*aphA6*	CP072299
KSK7	2020	Respiratory	India	622	p1KSK7	218105	-	TraHY	-	*mph/msr(E)*, *armA*, *sul1*, *bla*$_{PER-7}$, *sul1*, *cmlA1*, *arr-3*, *ant(3'')-la*, *sul2*, *strAB*	CP072276
					p2KSK7	68225	RP-T1	TraLEKBVCWUNFHG	-	*aphA6*	CP072277
					p3KSK7	7540	R3-T1	-	-	-	CP072278
					p4KSK7	5679	-	TraD	MobAL	*aphA6*	CP072279
KSK18	2020	Respiratory	India	622	p1KSK18	218105	-	TraHY	-	*mph/msr(E)*, *armA*, *sul1*, *bla*$_{PER-7}$, *sul1*, *cmlA1*, *arr-3*, *ant(3'')-la*, *sul2*, *strAB*	CP072291
					p2KSK18	68225	RP-T1	TraLEKBVCWUNFHG	-	*aphA6*	CP072292
					p3KSK18	7540	R3-T1	-	-	-	CP072293
					p4KSK18	5679	-	TraD	MobAL	*aphA6*	CP072294
KSK20	2020	Respiratory	India	622	p1KSK20	218105	-	TraHY	-	*mph/msr(E)*, *armA*, *sul1*, *bla*$_{PER-7}$, *sul1*, *cmlA1*, *arr-3*, *ant(3'')-la*, *sul2*, *strAB*	CP072301
					p2KSK20	68309	RP-T1	TraLEKBVCWUNFHG	-	*aphA6*	CP072302
					p3KSK20	7540	R3-T1	-	-	-	CP072303
					p4KSK20	5679	-	TraD	MobAL	*aphA6*	CP072304
AR_0070[a]	-	-	-	32	pAR_0070-1	105127	R3-T3	-	-	-	CP027182
					pAR_0070-2	73018	R3-T4	-	MobAL	-	CP027180
					pAR_0070-3	39535	R3-T19	-	-	*bla*$_{OXA-58}$ (3x)	CP027179
					pAR_0070-4	6078	-	-	MobAC	*aadB*	CP027181
Ab-B004d-c	2016	Sputum	Ghana	103	pAb-B004d-c_1	48239	R3-T20	-	MobC	*dfrA1*, *mph/msr(E)*, *tet39*, *aac(3)-lld*	CP051876
					pAb-B004d-c_2	8495	R3-T10	-	-	-	CP051877
					pAb-B004d-c_3	8215	-	TraD	MobAL	*bla*$_{OXA-23}$	CP051878
					pAb-B004d-c_4	2697	R1-T3	-	-	-	CP051879
KSK6	2020	Respiratory	India	622	p1KSK6	218105	-	TraH Y	-	*mph/msr(E)*, *armA*, *sul1*, *bla*$_{PER-7}$, *sul1*, *cmlA1*, *arr-3*, *ant(3'')-la*, *sul2*, *strAB*	CP072271
					p2KSK6	68224	RP-T1	TraLEKBVCWUNFHG	-	*aphA6*	CP072272
					p3KSK6	7540	R3-T1	-	-	-	CP072273
					p4KSK6	5679	-	TraD	MobAL	*aphA6*	CP072274

KSK2	2020	Respiratory	India	622	p1KSK2	218105	-	TraHY	-	*mph/msr(E)*, *armA*, *sul1*, $bla_{\text{PER-7}}$, *sul1*, *cmlA1*, *arr-3*, *ant(3")-la*, *sul2*, *strAB*	CP072399
					p2KSK2	87529	RP-T1	TraLEKBVCWUNFHG	-	$bla_{\text{OXA-23}}$, *aphA6*	CP072400
					p3KSK2	7540	R3-T1	-	-	-	CP072401
					p4KSK2	5679	-	TraD	MobAL	*aphA6*	CP072402
KSK11	2020	Respiratory	India	622	p1KSK11	218105	-	TraHY	-	*mph/msr(E)*, *armA*, *sul1*, $bla_{\text{PER-7}}$, *sul1*, *cmlA1*, *arr-3*, *ant(3")-la*, *sul2*, *strAB*	CP072286
					p2KSK11	87529	RP-T1	TraLEKBVCWUNFHG	-	$bla_{\text{OXA-23}}$, *aphA6*	CP072287
					p3KSK11	7540	R3-T1	-	-	-	CP072288
					p4KSK11	5679	-	TraD	MobAL	*aphA6*	CP072289
AR_0052[a]	-	-	-	32	pAR_0052-1	105128	R3-T3	-	-	-	CP027185
					pAR_0052-2	60699	R3-T19	-	-	$bla_{\text{OXA-58}}$ (4x)	CP027184
					pAR_0052-3	38505	R3-T4	-	MobAL	-	CP027186
					pAR_0052-4	6078	-	-	MobA	*aadB*	CP027187
D36	2008	Wound	Australia	81	pD36-4	47457	R3-T25, R3-T63	-	MobC	*sul2*, *aphA1*	CP012956
					pD36-3	9276	R3-T1	-	-	-	CP012955
					pD36-2	6078	-	-	MobAC	*aadB*	CP012954
					pD36-1	4754	-	-	MobAC	-	CP012953
KSK1	2020	Respiratory	India	622	p1KSK1	218105	-	TraHY	-	*mph/msr(E)*, *armA*, *sul1*, $bla_{\text{PER-7}}$, *sul1*, *cmlA1*, *arr-3*, *ant(3")-la*, *sul2*, *strAB*	CP072123
					p2KSK1	87529	RP-T1	TraLEKBVCWUNFHG	-	$bla_{\text{OXA-23}}$, *aphA6*	CP072124
					p3KSK1	7540	R3-T1	-	-	-	CP072125
					p4KSK1	5679	-	TraD	MobAL	*aphA6*	CP072126
NY5301	2018	Respiratory	China	2	pNY5301-1	110967	R3-T3	-	-	-	CP094284
					pNY5301-2	71194	RP-T1	TraLEKBVCWUNFHG	-	-	CP094285
					pNY5301-3	8731	R3-T1	-	-	-	CP094286
					pNY5301-4	2762	R3-T30	-	-	-	CP094287
MRSN 56	2010	Hip	USA	1	pMRSN56-4	8731	R3-T1	-	-	-	CP080456
					pMRSN56-3	6772	RP-T4	-	MobAC	-	CP080455
					pMRSN56-2	2725	R1-T1	-	-	-	CP080454
					pMRSN56-1	2178	R1-T6	-	-	-	CP080453
D46	2010	UTI	Australia	25	pD46-4	208004	r3-T60	TraYWCUONMIJ	-	*tet(B), sul2, mph/msr(E), strAB*	CP048135
					pD46-3	74916	RP-T1	TraAEKBVCWUNFHG, TrwBC	-	*aphA6*	CP048134
					pD46-2	8731	R3-T1	-	-	-	CP048133
					pD46-1	6078	-	-	MobAC	*ant(2")*	CP048132
DA33098	-	-	Spain	2	pDA33098-108	108151	R3-T9	-	-	-	CP029570
					pDA33098-71	71234	RP-T1	TraLEKBVCWNFHG	-	*aphA6*	CP029571
					pDA33098-9	8963	R3-T2	-	-	$bla_{\text{OXA-24}}$	CP029573
					pDA33098-9-2	8771	R3-T8	-	MobAL	$bla_{\text{OXA-24}}$	CP029572
36-1512	2013	Respiratory	Russia	203	p1.36-1512	113330	R3-T3	-	-	*sul2*	CP059387
					p2.36-1512	9458	R3-T13	-	MobAL	-	CP059388
					p3.36-1512	5688	R3-T2	-	-	-	CP059389
					p4.36-1512	4721	RP-T5	-	MobC	-	CP059390
ABNIH28	2016	Wastewater	USA	1543	pABA-2f10	130044	R3-T21	-	-	-	CP026129
					pABA-1fe1	110754	R3-T3	-	-	-	CP026128
					pNDM-0285	39359	-	TraMADC, TrbL	-	*ble-MBL*, $bla_{\text{NDM-1}}$, *aph(3')-Vl*	CP026127
					pABA-6973	11305	R3-T13	-	-	-	CP026126

Res13-Abat-PEA21-P4-01-A	2018	Swab	Canada	novel	pRes13-Abat_1[a]	113139	R3-T3	-	-	-	CP062920
					pRes13-Abat_2[a]	72831	RP-T1	TraLEKBVCWUNFHG	-	-	CP062921
					pRes13-Abat_3[a]	14288	R3-T29	-	-	-	CP062922
					pRes13-Abat_4[a]	5242	R3-T15	-	-	-	CP062923
AYE	2001	Pneumonia and UTI	France	1	p3ABAYE	94413	R3-T7	-	-	-	CU459140
					p2ABAYE	9661	R4-T1	-	-	-	CU459138
					p1ABAYE	5644	R3-T18	-	MobS	-	CU459137
					p4ABAYE	2726	R1-T1	-	-	-	CU459139
VB11737[b]	2019	Blood	India	2	pVB11737_1	70712	RP-T1	TraLEKBVCWUNFHG, TrbC	-	*aph(3')-6*	CP050392
					pVB11737_2	8731	R3-T1	-	-	-	CP050393
					pVB11737_3	3383	-	-	-	-	CP050394
					pVB11737_4	2637	-	-	-	-	CP050395

[a] Named here

[b] Strain was listed as containing 8 plasmids. However, the sequence of 4 plasmids (pVB11737_5, pVB11737_6, pVB11737_7 and pVB11737_8) only include internal fragments of IS or a resistance gene and hence cant represent plasmids.

```
MCR-4.7      MISRFKTLSVNQFTFITALFYVAIFNLPLFGIVRKGIEKQPEVDPLFIASMPLFLTFALS   60
MCR-4.3      MISRFKTLSVNQFTFITALFYVAIFNLPLFGIVRKGIEKQPEVDPLFIASMPLFLTFALS   60
MCR-4.6      MISRFKTLSVNQFTFITALFYVAIFNLPLFGIVRKGIEKQPEVDPLFIASMPLFLTFALS   60
MCR-4.5      MISRFKTLSVNQFTFITALFYVAIFNLPLFGIVRKGIEKQPEVDPLFIASMPLFLTFALS   60
MCR-4.4      MISRFKTLSVNQFTFITALFYVAIFNLPLFGIVRKGIEKQPEVDPLFIASMPLFLTFALS   60
MCR-4.1      MISRFKTLSVNQFTFITALFYVAIFNLPLFGIVRKGIEKQPEVDPLFIASMPLFLTFALS   60
Mcr-4.2      MISRFKTLSVNQFTFITALFYVAIFNLPLFGIVRKGIEKQPEVDPLFIASMPLFLTFALS   60
                                                            Pro110Leu
MCR-4.7      FLFSIFTVKYLLKPFFIVLTLLSSSVFFAAYQYNVVFDYGMIENTFQTHPAEALMYVNLA   120
MCR-4.3      FLFSIFTVKYLLKPFFIVLTLLSSSVFFAAYQYNVVFDYGMIENTFQTHPAEALMYVNLA   120
MCR-4.6      FLFSIFTVKYLLKPFFIVLTLLSSSVFFAAYQYNVVFDYGMIENTFQTHPAEALMYVNLA   120
MCR-4.5      FLFSIFTVKYLLKPFFIVLTLLSSSVFFAAYQYNVVFDYGMIENTFQTHLAEALMYVNLA   120
MCR-4.4      FLFSIFTVKYLLKPFFIVLTLLSSSVFFAAYQYNVVFDYGMIENTFQTHPAEALMYVNLA   120
MCR-4.1      FLFSIFTVKYLLKPFFIVLTLLSSSVFFAAYQYNVVFDYGMIENTFQTHPAEALMYVNLA   120
Mcr-4.2      FLFSIFTVKYLLKPFFIVLTLLSSSVFFAAYQYNVVFDYGMIENTFQTHPAEALMYVNLA   120
                                                                   Val179Gly
MCR-4.7      SITNLLLTGLLPSYLIYKADIHYQPFFKELLHKLAFMLLMFVGIGIVAFFYYQDYAAFGR   180
MCR-4.3      SITNLLLTGLLPSYLIYKADIHYQPFFKELLHKLAFMLLMFVGIGIVAFFYYQDYAAFGR   180
MCR-4.6      SITNLLLTGLLPSYLIYKADIHYQPFFKELLHKLAFMLLMFVGIGIVAFFYYQDYAAFVR   180
MCR-4.5      SITNLLLTGLLPSYLIYKADIHYQPFFKELLHKLAFMLLMFVGIGIVAFFYYQDYAAFVR   180
MCR-4.4      SITNLLLTGLLPSYLIYKADIHYQPFFKELLHKLAFMLLMFVGIGIVAFFYYQDYAAFVR   180
MCR-4.1      SITNLLLTGLLPSYLIYKADIHYQPFFKELLHKLAFMLLMFVGIGIVAFFYYQDYAAFVR   180
Mcr-4.2      SITNLLLTGLLPSYLIYKADIHYQPFFKELLHKLAFMLLMFVGIGIVAFFYYQDYAAFVR   180
                                   His205Asn                     Val236Phe
MCR-4.7      NNSELRRYIVPTYFVSSASKYLNEHYLQTPMEYQQLGLDAKNASRNPNTKPNLLVFVVGE   240
MCR-4.3      NNSELRRYIVPTYFVSSASKYLNEHYLQTPMEYQQLGLDAKNASRNPNTKPNLLVFVVGE   240
MCR-4.6      NNSELRRYIVPTYFVSSASKYLNEHYLQTPMEYQQLGLDAKNASRNPNTKPNLLVFVVGE   240
MCR-4.5      NNSELRRYIVPTYFVSSASKYLNEHYLQTPMEYQQLGLDAKNASRNPNTKPNLLVVVVGE   240
MCR-4.4      NNSELRRYIVPTYFVSSASKYLNENYLQTPMEYQQLGLDAKNASRNPNTKPNLLVVVVGE   240
MCR-4.1      NNSELRRYIVPTYFVSSASKYLNEHYLQTPMEYQQLGLDAKNASRNPNTKPNLLVVVVGE   240
Mcr-4.2      NNSELRRYIVPTYFVSSASKYLNEHYLQTPMEYQQLGLDAKNASRNPNTKPNLLVVVVGE   240

MCR-4.7      TARSMSYQYYGYNKPTNAHTQNQGLIAFNDTSSCGTATAVSLPCMFSRMGRADYDPRRAN   300
MCR-4.3      TARSMSYQYYGYNKPTNAHTQNQGLIAFNDTSSCGTATAVSLPCMFSRMGRADYDPRRAN   300
MCR-4.6      TARSMSYQYYGYNKPTNAHTQNQGLIAFNDTSSCGTATAVSLPCMFSRMGRADYDPRRAN   300
MCR-4.5      TARSMSYQYYGYNKPTNAHTQNQGLIAFNDTSSCGTATAVSLPCMFSRMGRADYDPRRAN   300
MCR-4.4      TARSMSYQYYGYNKPTNAHTQNQGLIAFNDTSSCGTATAVSLPCMFSRMGRADYDPRRAN   300
MCR-4.1      TARSMSYQYYGYNKPTNAHTQNQGLIAFNDTSSCGTATAVSLPCMFSRMGRADYDPRRAN   300
Mcr-4.2      TARSMSYQYYGYNKPTNAHTQNQGLIAFNDTSSCGTATAVSLPCMFSRMGRADYDPRRAN   300
                                        Gln331Arg
MCR-4.7      AQDTVIDVLSHSGIKVQWFDNDSGCKGVCDRVENLTIDLKSDPKLCSGQYCFDQVLLNKL   360
MCR-4.3      AQDTVIDVLSHSGIKVQWFDNDSGCKGVCDQVENLTIDLKSDPKLCSGQYCFDQVLLNKL   360
MCR-4.6      AQDTVIDVLSHSGIKVQWFDNDSGCKGVCDQVENLTIDLKSDPKLCSGQYCFDQVLLNKL   360
MCR-4.5      AQDTVIDVLSHSGIKVQWFDNDSGCKGVCDRVENLTIDLKSDPKLCSGQYCFDQVLLNKL   360
MCR-4.4      AQDTVIDVLSHSGIKVQWFDNDSGCKGVCDRVENLTIDLKSDPKLCSGQYCFDQVLLNKL   360
MCR-4.1      AQDTVIDVLSHSGIKVQWFDNDSGCKGVCDQVENLTIDLKSDPKLCSGQYCFDQVLLNKL   360
Mcr-4.2      AQDTVIDVLSHSGIKVQWFDNDSGCKGVCDRVENLTIDLKSDPKLCSGQYCFDQVLLNKL   360

MCR-4.7      DKILAVAPSQDTVIFLHIIGSHGPTYYLRYPPEHRKFIPDCPRSDIQNCSQEELINTYDN   420
MCR-4.3      DKILAVAPSQDTVIFLHIIGSHGPTYYLRYPPEHRKFIPDCPRSDIQNCSQEELINTYDN   420
MCR-4.6      DKILAVAPSQDTVIFLHIIGSHGPTYYLRYPPEHRKFIPDCPRSDIQNCSQEELINTYDN   420
MCR-4.5      DKILAVAPSQDTVIFLHIIGSHGPTYYLRYPPEHRKFIPDCPRSDIQNCSQEELINTYDN   420
MCR-4.4      DKILAVAPSQDTVIFLHIIGSHGPTYYLRYPPEHRKFIPDCPRSDIQNCSQEELINTYDN   420
MCR-4.1      DKILAVAPSQDTVIFLHIIGSHGPTYYLRYPPEHRKFIPDCPRSDIQNCSQEELINTYDN   420
Mcr-4.2      DKILAVAPSQDTVIFLHIIGSHGPTYYLRYPPEHRKFIPDCPRSDIQNCSQEELINTYDN   420

MCR-4.7      TILYTDFILSEVVNKLKGKQDMFDTAMLYLSDHGESLGEKGMYLHGAPYSIAPKEQTSVP   480
MCR-4.3      TILYTDFILSEVVNKLKGKQDMFDTAMLYLSDHGESLGEKGMYLHGAPYSIAPKEQTSVP   480
MCR-4.6      TILYTDFILSEVVNKLKGKQDMFDTAMLYLSDHGESLGEKGMYLHGAPYSIAPKEQTSVP   480
MCR-4.5      TILYTDFILSEVVNKLKGKQDMFDTAMLYLSDHGESLGEKGMYLHGAPYSIAPKEQTSVP   480
MCR-4.4      TILYTDFILSEVVNKLKGKQDMFDTAMLYLSDHGESLGEKGMYLHGAPYSIAPKEQTSVP   480
MCR-4.1      TILYTDFILSEVVNKLKGKQDMFDTAMLYLSDHGESLGEKGMYLHGAPYSIAPKEQTSVP   480
Mcr-4.2      TILYTDFILSEVVNKLKGKQDMFDTAMLYLSDHGESLGEKGMYLHGAPYSIAPKEQTSVP   480

               Val485Ile
MCR-4.7      MLAWISNDFSQDNQLNMTCVAQRAEQGGFSHDNLFDSLLGLMNVKTTVYQSQLDIFAPCR   540
MCR-4.3      MLAWVSNDFSQDNQLNMTCVAQRAEQGGFSHDNLFDSLLGLMNVKTTVYQSQLDIFAPCR   540
MCR-4.6      MLAWVSNDFSQDNQLNMTCVAQRAEQGGFSHDNLFDSLLGLMNVKTTVYQSQLDIFAPCR   540
MCR-4.5      MLAWVSNDFSQDNQLNMTCVAQRAEQGGFSHDNLFDSLLGLMNVKTTVYQSQLDIFAPCR   540
MCR-4.4      MLAWVSNDFSQDNQLNMTCVAQRAEQGGFSHDNLFDSLLGLMNVKTTVYQSQLDIFAPCR   540
MCR-4.1      MLAWVSNDFSQDNQLNMTCVAQRAEQGGFSHDNLFDSLLGLMNVKTTVYQSQLDIFAPCR   540
Mcr-4.2      MLAWVSNDFSQDNQLNMTCVAQRAEQGGFSHDNLFDSLLGLMNVKTTVYQSQLDIFAPCR   540

MCR-4.7      Y      541
MCR-4.3      Y      541
MCR-4.6      Y      541
MCR-4.5      Y      541
MCR-4.4      Y      541
MCR-4.1      Y      541
Mcr-4.2      Y      541
```

Figure S1. Sequence alignment showing variation in amino acid sequences with respect to MCR-4.1 using Clustal Omega. The MCR-4 allele sequences were retrieved from NCBI with the following accession numbers: MCR-4.1(WP_099156046.1), MCR-4.2(AXN76074.1), MCR-4.3(WP_011638903.1), MCR-4.4(WP_109545055.1), MCR-4.5(WP_109545054.1), MCR-4.6(WP_116786828.1) and MCR-4.7(p8E072658)

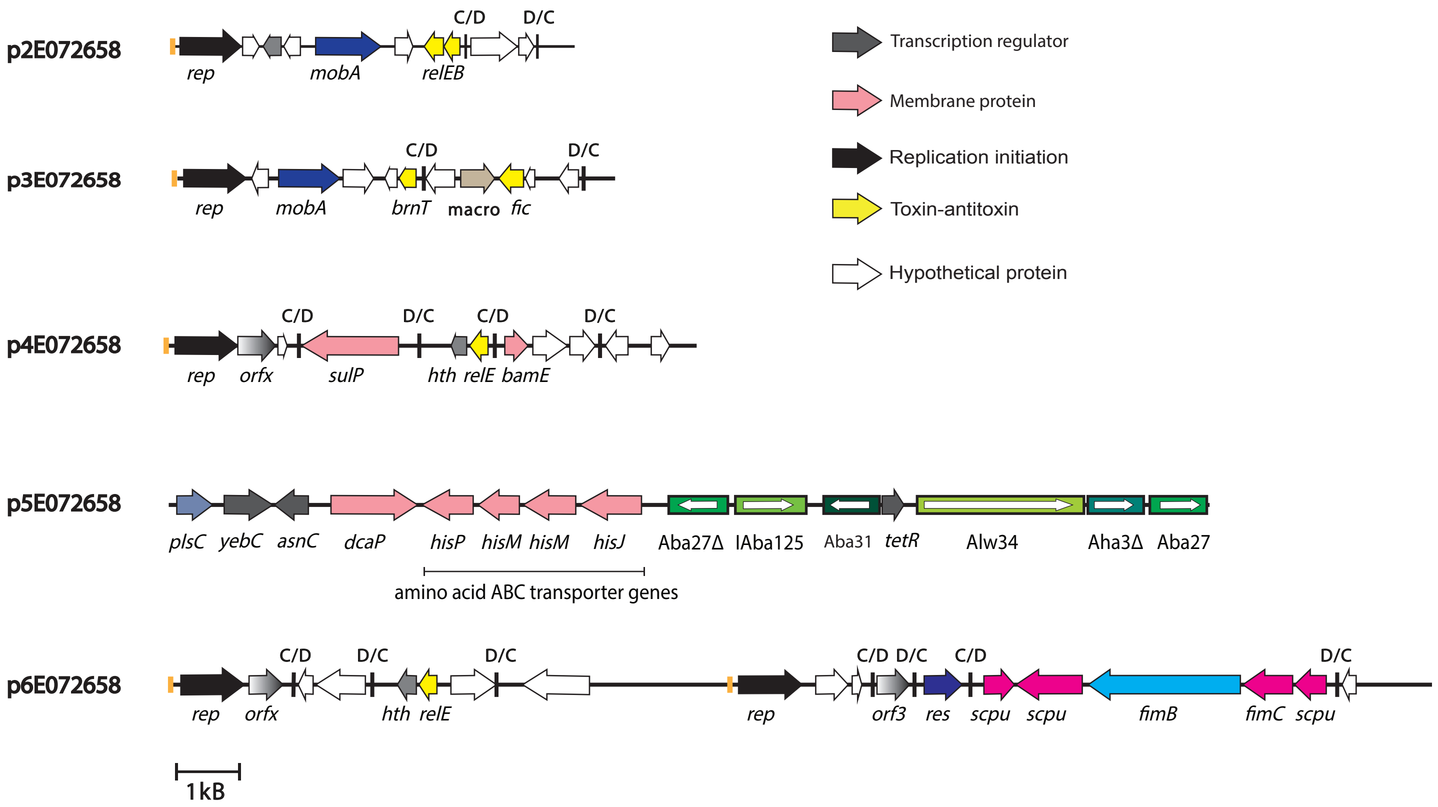

Figure S2. Genetic structure of cryptic plasmids found in E-072658.
Arrows indicate the extent and orientation of genes. Replication genes are coloured black and Insertion sequences are shown using green boxes.
